# Rationale and Methods for the National Tuberculosis Genotyping and Surveillance Network

**DOI:** 10.3201/eid0811.020408

**Published:** 2002-11

**Authors:** Kenneth G. Castro, Harold W. Jaffe

**Affiliations:** * Centers for Disease Control and Prevention, Atlanta, Georgia, USA

**Keywords:** Tuberculosis, Mycobacterium tuberculosis, genetic typing, genotyping, molecular epidemiology

## Abstract

Our understanding of tuberculosis (TB) transmission dynamics has been refined by genotyping of *Mycobacterium tuberculosis* strains. The National Tuberculosis Genotyping and Surveillance Network was designed and implemented to systematically evaluate the role of genotyping technology in improving TB prevention and control activities. Genotyping proved a useful adjunct to investigations of outbreaks, unusual clusters, and laboratory cross-contamination.

“In the future, the battle against this plague of mankind will not just be concerned with an uncertain something but with a tangible parasite, about whose characteristics a great deal is known and can be explored.”—Robert Koch, 1882

Molecular genetic typing (or genotyping) of *Mycobacterium tuberculosis* strains has revolutionized the field of tuberculosis (TB) research, prevention, and control (1–3). The subtypes characterized by molecular genetic typing methods provide a greater power and ability to differentiate strains than previous methods, such as comparisons of patterns of drug resistance or phage-typing (4,5). When molecular genotyping technology is applied to outbreaks or unusual clusters of disease, persons sharing *M. tuberculosis* strains can be identified, which can lead to important clues about the pattern and dynamics of transmission.

Methods of molecular genotyping have been increasingly applied to the epidemiology of TB. Because of its natural history, the transmission of *M. tuberculosis* is difficult to study; *M. tuberculosis* is spread by airborne droplets of respiratory secretions expelled by an infectious person to a susceptible host, who may or may not be known to the source (6). The bacterium can remain latent as an asymptomatic infection for years, and the source of such infections can be difficult to ascertain. Thus, the places and persons involved in a chain of transmission may be puzzling to identify or exclude. Molecular typing of *M. tuberculosis* adds important pieces to the construction of such a transmission puzzle; persons who harbor the same strain of *M. tuberculosis* are likely to have shared that strain in a common chain of transmission; by contrast, persons who are infected by unique and distinct strains were probably infected by means of a different exposure. Among its applications, genotyping has served to elucidate the poorly understood role of relapses and exogenous reinfection of persons with recurrent TB after cure. Several reports have relied on DNA genotyping to describe and document the occurrence of exogenous reinfection with distinct strains of *M. tuberculosis* as the cause of TB following successful treatment (7–9).

The usefulness of molecular typing was also confirmed in several epidemiologic investigations of HIV-associated multidrug-resistant TB outbreaks in hospices, hospitals, and prisons during the late 1980s and early 1990s and provided compelling evidence of institutional transmission (10–16). Unique features of these outbreaks included prolonged infectiousness of patients who were not recognized to harbor multidrug-resistant TB until months after their TB was diagnosed and the relatively rapid progression from latent infection to active TB disease in persons with HIV-associated immunosuppression. These data were also used to state the need to implement effective interventions to halt such outbreaks (17,18).

In addition to the use of DNA genotyping during outbreak investigations, the technology has been applied as a complementary tool to conventional methods in TB control (19–22). In two of the earliest studies conducted in the United States, one in San Francisco and one in New York City, the authors assumed that *M. tuberculosis* isolates with matching DNA fingerprints were epidemiologically related and represented recent transmission of *M. tuberculosis* among the patients involved (i.e., within 2 years before diagnosis) (19–20). In these studies, 30% to 40% of the patients had *M. tuberculosis* isolates with DNA fingerprint patterns that matched at least one other isolate. This finding led the authors to conclude that as many as 40% of TB cases in these two cities were the result of recent transmission and that TB control practices in San Francisco and New York were not effectively decreasing *M. tuberculosis* transmission. The observations in these two reports were useful and innovative. However, the findings could not be generalized to other geographic areas in the United States because the study populations were exclusively urban residents, a large proportion were HIV infected, and detailed epidemiologic information was incomplete for either study, which limited the ability to corroborate actual contacts or exposures among the TB patients studied.

A separate study evaluated the use of DNA genotyping in TB patients from a large, rural population in the state of Arkansas (21). Analysis of *M. tuberculosis* isolates from TB patients for a 2-year period (1992–1993) found that more than half the isolates matched at least one other patient isolate. Epidemiologic investigation of the patients with matching *M. tuberculosis* isolates (i.e., clustered cases) revealed 24 persons who had documented latent TB infection or active TB many years in the past but which produced disease with matching isolates at the time of the study. In five of these patients, a remote epidemiologic connection (i.e., common exposures to a person with TB) was identified that occurred 20–25 years earlier. The authors also reported that some TB patients had isolates showing identical specific patterns, yet their geographic, social, and medical histories were so disparate that transmission among them was highly unlikely. Further investigation by genotyping with an independent method revealed that the capacity of the IS*6110* method to differentiate strains was roughly proportional to the number of bands present in the original fingerprint pattern. Thus, isolates with a low number of bands (e.g., fewer than five) required a second method for appropriate differentiation. These findings suggested the need for additional assessments and evaluations of emerging assumptions in the interpretation of DNA genotyping of *M. tuberculosis*.

The fruitful use of DNA genotyping to confirm and refine our understanding of *M. tuberculosis* transmission in outbreaks provided the major impetus to evaluate the use of this technology in other settings and to determine its broader application as a tool in TB prevention and control. Specifically, we sought to assess the usefulness of this technology in searching for unidentified outbreaks, identifying risk factors for TB at the population level, and identifying and monitoring laboratory cross-contamination (23). Consequently, the Centers for Disease Control and Prevention (CDC) funded six laboratories in 1993 to develop regional databases of DNA genotype fingerprint patterns and undertake regional molecular epidemiologic studies. This genotyping network was expanded in 1996 to include sentinel surveillance sampling. Ultimately, the National Tuberculosis Genotyping and Surveillance Network comprised CDC, seven laboratories, and seven sentinel surveillance sites in the United States.

## Objectives and Composition of the National Tuberculosis Genotyping and Surveillance Network

Successful applicants for a cooperative agreement with CDC formed part of the genotyping network. The following six potential objectives were to be the focus of activities by the network:

### 1. Determine the Relative Frequency of *M. tuberculosis* Strains on the Basis of DNA Fingerprint Patterns by Using the IS*6110* Method in Specific Geographic Areas

This determination was meant to characterize the diversity of strains in any one area or region and allow for more accurate interpretation of results of DNA fingerprint analysis.

### 2. Determine the Extent of Spread of Related *M. tuberculosis* Strains in Communities

As a secondary objective, the identification of common and potentially more transmissible strains could aid in the study of *M. tuberculosis* pathogenesis and host immunity.

### 3. Describe the Geographic Mobility of Related *M. tuberculosis* Strains and the Mode in Which Strains Spread

The characterization of places and activities involved in potential transmission could enable TB control programs to design interventions accordingly.

### 4. Determine the Relatedness of *M. tuberculosis* Isolates in Patients Who Are Identified as Being a High Risk for TB through Conventional Epidemiologic Studies

DNA fingerprint clustering of isolates among groups at high risk could represent a relatively more specific marker of recent transmission when compared to clustering identified in the general population.

### 5. Develop the Capacity of Local TB Controllers To Identify Patients with Related *M. Tuberculosis* Organisms Who Deserve Careful Consideration and Investigation To Identify Ongoing Transmission

A secondary objective was to assess the role of fingerprinting in helping to prioritize and focus contact investigations.

### 6. Assess Use of DNA Fingerprinting Analyses in Guiding TB Control Activities, such as Targeted Testing and Treatment of Latent TB Infection and Monitoring Possibilities for Transmission in Congregate Settings such as Hospitals and Prisons

Federal funds were provided for the TB genotyping network to establish a core set of databases at each of the laboratories and sentinel surveillance sites and a national database at CDC. The laboratory databases included computerized images of DNA fingerprint patterns from all *M. tuberculosis* isolates analyzed for their region and for all isolates analyzed for their sentinel surveillance site. The databases at the sentinel sites included a record for each sentinel area resident who was diagnosed with culture-positive TB. The record contained information collected as part of routine TB national surveillance activities; the identification of source or secondary cases, if known; and the DNA fingerprint pattern designation. Routine surveillance for TB included information for each patient concerning demographics, social and occupational risk factors for TB; clinical and radiologic details of disease; culture, strain, and histology results; susceptibility testing of isolates; and antibiotic treatment regimens and clinical outcome. At CDC, DNA fingerprint images, surveillance, and epidemiologic information were combined from all laboratories and sites to create national databases of sentinel site patients and a library of all unique DNA fingerprint patterns among isolates from sentinel surveillance site patients.

## Sentinel Surveillance Sites and Regional Laboratories

Sentinel surveillance sites in the network included the states of Arkansas, Maryland, Massachusetts, Michigan, and New Jersey; six counties in California (Alameda, Contra Costa, Marin, San Mateo, Santa Clara, and Solano); and four counties in Texas (Dallas, Tarrant, Cameron, and Hidalgo). All patients within those areas were included on a prospective basis. The sentinel surveillance sites were selected on the basis of applications by state and large-city departments of health and by characteristics of the proposed sentinel populations.

The following criteria were used to evaluate sites applying through competitive proposals: 1) understand the use of *M. tuberculosis* DNA fingerprinting in the epidemiology of TB; 2) report at least 250 TB cases per year, and submit one *M. tuberculosis* isolate for ∃75% of culture-positive patients in their areas for DNA fingerprinting; 3) conduct active surveillance of TB cases; 4) review information from the national TB surveillance database and make every effort to ensure that data are complete; 5) establish and maintain a surveillance site database; and 6) maintain records of activities performed as part of the genotyping network.

The network sentinel surveillance site relied on local mycobacteriology and hospital infection control records for all facilities in the surveillance site areas for case finding. Other sources of information included hospital IDC-9 discharge codes for TB, pharmacy records for prescriptions of a combination of two or more anti-TB drugs, coroners’ records that showed TB as a diagnosis, and AIDS surveillance reports that indicated a diagnosis of TB. Sentinel surveillance site personnel reviewed the information in their national surveillance database to ensure that all information was complete to the extent possible.

The regional laboratories were also selected on a competitive basis. They were responsible for providing DNA fingerprint analysis of *M. tuberculosis* isolates from health departments in their region and for their assigned sentinel surveillance site. Regions were assigned to laboratories on the basis of history of their work and approximate numbers of TB patients in the regions. Each culture-positive TB case normally reported for national TB surveillance (on the form, Report of a Verified Case of Tuberculosis) within the sentinel site area was included as a sentinel surveillance case. An isolate from each culture-positive TB patient was sent for DNA fingerprinting at the designated regional DNA fingerprinting laboratory. *M. tuberculosis* isolates were shipped from sentinel sites to laboratories in approved shipping containers that were appropriately labeled and handled in accordance with bio-safety level 3 conditions. Subcultures of isolates were to be stored by the regional laboratories at –70°C in duplicate indefinitely.

At the regional laboratories, images of individual DNA fingerprint patterns of *M. tuberculosis* isolates were generated by using standardized procedures for DNA extraction, purification, digestion, electrophoresis, hybridization, and computerization. Individual images were electronically transmitted to CDC to be assigned a national DNA fingerprint designation. Each submitted fingerprint pattern was compared to previously submitted patterns. Unique patterns were added to the database and assigned consecutive five-digit numbers as their national designations. Results of analysis of isolates and DNA fingerprint designations were reported back to the regional DNA fingerprinting laboratories on a regular basis. The results of DNA fingerprint analysis of sentinel surveillance TB case isolates, including the national DNA fingerprint database pattern designations, were transmitted from the regional laboratories to the sentinel surveillance sites.

This special issue of Emerging Infectious Diseases contains the reports of the various analyses resulting from this highly productive collaboration of the National Tuberculosis Genotyping and Surveillance Network. The results represent a remarkable accomplishment and provide the scientific basis for future potential applications of DNA genotyping as part of population-based TB prevention and control activities in the United States. The results also highlight both the strengths and limitations of DNA genotyping as an adjunct to TB outbreak and contact investigations and assessments of laboratory cross-contamination. Although the network has been very productive, additional technologic advances are necessary as well as improvements in the understanding and use of principles and practices from other disciplines, such as social network analyses, before we can reliably obtain real-time laboratory results and improve our understanding of events facilitating the transmission of *M. tuberculosis* in modern societies. Such a comprehensive approach that combines both basic and operational research must be supported so that our efforts will ultimately result in the elimination of TB (24).

## References

[1] Cave MD, Eisenach KD, McDermott PF, Bates JH, Crawford JT. IS*6110*: Conservation of sequence in the *Mycobacterium tuberculosis* complex and its utilization in DNA fingerprinting. Mol Cell Probes. 1991;5:73–80. 10.1016/0890-8508(91)90040-Q 1673228

[2] van Embden JDA, Cave MD, Crawford JT, Dale JW, Eisenach KD, Giquel B, Strain identification of *Mycobacterium tuberculosis* by DNA fingerprinting: recommendations for a standardized methodology. J Clin Microbiol. 1993;31:406–9. 8381814 10.1128/jcm.31.2.406-409.1993 PMC262774

[3] Mazurek GH, Cave MD, Eisenach KD, Wallace RJ, Bates JH, Crawford JT. Chromosomal DNA fingerprint patterns produced with IS*6110* as strain-specific markers for epidemiologic study of tuberculosis. J Clin Microbiol. 1991;29:2030–1. 1663520 10.1128/jcm.29.9.2030-2033.1991 PMC270253

[4] Bates JH, Mitchinson DA. Geographic distribution of bacteriophage types of *Mycobacterium tuberculosis.* Am Rev Respir Dis. 1969;100:189–93. 4979744 10.1164/arrd.1969.100.2.189

[5] Rado TA, Bates JH, Engel HW, Mankiewicz E, Murohashi T, Mizuguchi Y, World Health Organization studies on bacteriophage typing of mycobacteria: subdivision of the species *Mycobacterium tuberculosis.* Am Rev Respir Dis. 1975;111:459–68. 804840 10.1164/arrd.1975.111.4.459

[6] Riley R, Mills C, Nyka W. Aerial dissemination of pulmonary tuberculosis—a two-year study of contagion in a tuberculosis ward. Am J Hyg. 1959;70:185–96. 10.1093/oxfordjournals.aje.a117542 7785671

[7] Small PM, Shafer RW, Hopewell PC, Singh SP, Murphy MJ, Desmond E, Exogenous reinfection with multidrug-resistant *Mycobacterium tuberculosis* in patients with advanced HIV infection. N Engl J Med. 1993;328:1137–44. 10.1056/NEJM199304223281601 8096066

[8] van Rie A, Warren R, Richardson M, Victor TC, Gie RP, Enarson DA, . Exogenous reinfection as a cause of recurrent tuberculosis after curative treatment. N Engl J Med. 1999;341:1174–9. 10.1056/NEJM199910143411602 10519895

[9] Sonnenberg R, Murray J, Gllynn JR, Shearer S, Kambashi B, Godfrey-Faussett P. HIV-1 and recurrence, relapse, and reinfection of tuberculosis after cure: a cohort study in South African miners. Lancet. 2001;358:1687–93. 10.1016/S0140-6736(01)06712-5 11728545

[10] Centers for Disease Control and Prevention. Transmission of multidrug-resistant tuberculosis among HIV-infected persons—Florida and New York, 1988-1991. MMWR Morb Mortal Wkly Rep. 1991;40:585–91. 1870559

[11] Daley CL, Small PM, Schecter GF, Schoolnick GK, McAdam RA, Jacobs WR Jr, An outbreak of tuberculosis with accelerated progression among persons infected with the human immunodeficiency virus. N Engl J Med. 1992;326:231–5. 1345800 10.1056/NEJM199201233260404

[12] Edlin BR, Tokars JI, Grieco MH, Crawford JT, Williams J, Sordillo EM, An outbreak of multidrug resistant tuberculosis among hospitalized patients with the acquired immunodeficiency syndrome. N Engl J Med. 1992;326:1514–21. 1304721 10.1056/NEJM199206043262302

[13] Beck-Sague C, Dooley SW, Hutton MD, Otten J, Breeden A, Crawford JT, Hospital outbreak of multidrug-resistant *Mycobacterium tuberculosis* infections. JAMA. 1992;268:1280–6. 10.1001/jama.268.10.1280 1507374

[14] Coronado VG, Beck-Sague CM, Hutton MD, Davis BJ, Nicholas P, Villareal C, Transmission of multidrug-resistant *Mycobacterium tuberculosis* among persons with human immunodeficiency virus infection in an urban hospital. J Infect Dis. 1993;168:1052–5. 8104226 10.1093/infdis/168.4.1052

[15] Centers for Disease Control and Prevention. Transmission of multidrug-resistant tuberculosis among immunocompromised persons in a correctional system—New York, 1991. MMWR Morb Mortal Wkly Rep. 1992;41:507–9. 1630427

[16] Valway SE, Richard SB, Kovakovich J, Greifinger RB, Crawford JT, Dooley SW, Outbreak of multidrug-resistant tuberculosis in a New York State Prison, 1991. Am J Epidemiol. 1994;140:113–22. 8023800 10.1093/oxfordjournals.aje.a117222

[17] Centers for Disease Control and Prevention. Guidelines for preventing the transmission of tuberculosis in health-care settings with special focus on HIV-related issues. MMWR Morb Mortal Wkly Rep. 1990;39(No. RR-1):1–29. 2175838

[18] Centers for Disease Control and Prevention. Guidelines for preventing the transmission of *Mycobacterium tuberculosis* in health-care facilities, 1994. MMWR Morb Mortal Wkly Rep. 1994;43(No. RR-13):1–132. 8602125

[19] Small PM, Hopewell PC, Singh SP, Paz A, Parsonnet J, Ruston DC, The epidemiology of tuberculosis in San Francisco—a population-based study using conventional and molecular methods. N Engl J Med. 1994;330:1703–9. 10.1056/NEJM199406163302402 7910661

[20] Alland D, Kalkut GE, Moss AR, McAdam RA, Hahn JA, Bosworth W, Transmission of tuberculosis in New York City—an analysis by DNA fingerprinting and conventional epidemiologic methods. N Engl J Med. 1994;330:1710–6. 10.1056/NEJM199406163302403 7993412

[21] Braden CR, Templeton GL, Cave MD, Valway S, Onorato IM, Castro KG, Interpretation of restriction fragment length polymorphism analysis of *Mycobacterium tuberculosis* isolates from a state with a large rural population. J Infect Dis. 1997;175:1446–52. 10.1086/516478 9180185

[22] van Deutekom H, Gerritsen JJ, van Soolingen D, van Ameijden EJ, van Embden JD, Coutinho RA. A molecular epidemiological approach to studying the transmission of tuberculosis in Amsterdam. Clin Infect Dis. 1997;25:1071–7. 10.1086/516072 9402360

[23] Braden CR, Templeton GL, Stead WW, Bates JH, Cave MD, Valway SE. Retrospective detection of laboratory cross-contamination of *Mycobacterium tuberculosis* cultures with use of DNA fingerprint analysis. Clin Infect Dis. 1997;24:35–40. 8994753 10.1093/clinids/24.1.35

[24] Institute of Medicine. Ending neglect: the elimination of tuberculosis in the United States. Washington: National Academy Press; 2000. p. 1–269.

